# Sequential induction chemotherapy plus intensity‐modulated radiotherapy versus concurrent chemoradiotherapy in locoregionally advanced nasopharyngeal carcinoma: the three‐year report of a phase II, single center, randomized, non‐inferiority trial

**DOI:** 10.1002/cam4.3936

**Published:** 2021-05-06

**Authors:** Zhining Yang, Zeman Cai, Qingxin Cai, Yingji Hong, Cuidai Zhang, Kaichun Huang, Zhixiong Lin, Mei Li

**Affiliations:** ^1^ Department of Radiation Oncology Cancer Hospital of Shantou University Medical College Shantou Guangdong China; ^2^ Shantou University Medical College Shantou Guangdong China

**Keywords:** cancer management, chemotherapy, clinical trials, nasopharyngeal carcinoma, neoadjuvant chemotherapy, radiotherapy

## Abstract

To compare the efficacy and safety of induction chemotherapy (IC) followed by intensity‐modulated radiotherapy (IMRT) alone versus concurrent CCRT in locoregionally advanced nasopharyngeal carcinoma (LA‐NPC). Patients with newly diagnosed stage III to IVB nasopharyngeal carcinoma (NPC) were randomized to receive IC plus IMRT (IC+RT arm), or concurrent chemotherapy plus IMRT (CCRT arm), using a random number table. Both treatment arms received the same chemotherapy regimen. The primary endpoint was progression‐free survival (PFS). Secondary end points included overall survival (OS), locoregional recurrence‐free survival (LRRFS), distant metastasis‐free survival (DMFS), treatment response, and acute treatment toxicities. From June 2013 to September 2018, a total of 204 patients histologically diagnosed with LA‐NPC were enrolled in the study, with 102 patients randomly assigned to each arm. After a median follow‐up duration of 45 months (range 4 to 84 months), the 3‐year PFS, OS, LRRFS and DMFS were 72.2%, 87.8%, 92.3%, and 82.7% in the IC+RT arm, compared with 82.6%, 92.8%, 94.7%, and 88.2% in the CCRT arm. No statistical difference for PFS, OS, LRRFS, DMFS, or treatment response was observed between the two arms (*p *> 0.05). The incidences of leukopenia (*p* = 0.008) and anemia (*p* = 0.015) were significantly higher in patients in the CCRT arm than those in the IC+RT arm. Compared to CCRT, IC plus IMRT alone provided similarly favorable treatment outcomes in terms of PFS, OS, LRRFS, and DMFS for patients with LA‐NPC, but resulted in fewer incidences of leukopenia and anemia.

## Graphical abstract

Concurrent chemoradiotherapy is the standard modality for locoregionally advanced nasopharyngeal carcinoma, accompanied with non‐negligible toxicities and poor compliance. The authors present comparable favorable treatment results in a randomize trial comparing induction chemotherapy plus IMRT versus CCRT. While induction chemotherapy plus IMRT had fewer incidences of leukopenia and anemia. The authors propose that induction chemotherapy plus IMRT is a promising option for locoregionally advanced nasopharyngeal carcinoma and further investigation to identify favorite individuals is warranted.

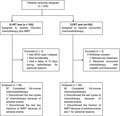

**Abbreviations**
AC: adjuvant chemotherapy
CCRT: concurrent chemoradiotherapy
DMFS: distant metastasis‐free survival
IC: induction chemotherapy
IMRT: intensity‐modulated radiotherapy
LA‐NPC: locoregionally advanced nasopharyngeal carcinoma
LRRFS: locoregional recurrence–free survival
NPC: nasopharyngeal carcinoma
OS: overall survival
PFS: progression‐free survival
PTV: planning target volume
RT: radiotherapy

## 1 INTRODUCTION

Nasopharyngeal carcinoma (NPC) has a geographical and ethnic variation in its distribution. There were 129079 new cases of NPC reported worldwide in 2018, with the highest incidence in South China.^1^ Platinum‐based concurrent chemoradiotherapy (CCRT) is considered the standard treatment for locoregionally advanced nasopharyngeal carcinoma (LA‐NPC), which accounts for more than 70% of the new cases of NPC^2^ and has an unsatisfying prognosis.^3^ Several randomized trials,^4^, ^5^, ^6^, ^7^, ^8^, ^9^, ^10^ as well as meta‐analyses,^11^, ^12^, ^13^ have demonstrated a superior treatment effect of CCRT over RT alone in LA‐NPC. However, the evidence has mostly been established on conventional radiotherapy. Further retrospective studies investigating CCRT with intensity‐modulated radiotherapy (IMRT) versus IMRT alone in LA‐NPC did not confirm the advantages of CCRT.^14^, ^15^ Moreover, the high incidence of treatment toxicities and poor compliance resulting from CCRT are also not negligible. Therefore, with IMRT currently being the mainstay technique for NPC, it is reasonable to question the superiority of CCRT and optimize the combination of chemotherapy and IMRT.

The addition of induction chemotherapy (IC) to CCRT has achieved encouraging outcomes for patients with LA‐NPC.^16^, ^17^, ^18^, ^19^ Previous studies showed that IC followed by IMRT alone provided comparable efficacy to IC plus CCRT,^20^, ^21^, ^22^ as well as CCRT with or without adjuvant chemotherapy (AC).^21^, ^23^, ^24^ Moreover, the toxicities of IC plus IMRT are significantly lower.^20^, ^22^, ^24^

Docetaxel plus cisplatin (TP) as an IC regimen for LA‐NPC has been shown by a phase 2 study.^25^ However, when given two cycles of TP concurrently with IMRT we observed a high incidence of severe toxicities and poor compliance in our practice. Thus, we changed the concurrent chemotherapy regimen to one cycle of docetaxel plus cisplatin and one cycle of cisplatin alone (TP/DDP) in our protocol and conducted this single center, randomized controlled, phase 2 non‐inferiority clinical trial to compare the efficacy and safety of sequential TP/DDP IC followed by IMRT alone versus CCRT for LA‐NPC. The trial protocol was approved by the institutional ethics committee and was registered at the Chinese Clinical Trial Registry with the registration number of ChiCTR‐TRC‐14004341.

## 2 MATERIALS AND METHODS

### 2.1 Patient eligibility and randomization assignment

Patients were deemed eligible if they met the following criteria: histologically proven nonkeratinizing NPC (including WHO type II and III disease); newly diagnosed stage III to IVB disease (7^th^ edition of the UICC/AJCC staging system^26^); age between 18 and 70 years; no history of treatment for cancer; an Eastern Cooperative Oncology Group performance status score of 0 to 2; adequate hematologic, hepatic and renal function; and absence of pregnancy, lactation, second malignancy, or severe coexisting disease.

Pretreatment evaluation included a complete patient history and physical examination, full hematology and biochemistry profiles, flexible nasopharyngoscopy, ECG, MRI or enhanced CT of the nasopharynx and neck, chest radiograph or CT, abdominal sonography, and bone scan. Eligible patients were required to provide written informed consent and then randomized to participate in the IC+RT arm, or CCRT arm using a random number table.

### 2.2 Chemotherapy

Eligible patients in both treatment arms received the same chemotherapy regimen of TP/DDP, which was administered as one cycle of TP (docetaxel 75 mg/m^2^ on Day 1 and cisplatin 25 mg/m^2^ per day from Day 1 to 3) and one cycle of DDP alone (cisplatin 25 mg/m^2^ per day from Day 1 to 3), given intravenously with an interval of 3 weeks.

Cisplatin was reduced by 25% if patients developed febrile neutropenia, grade 3 thrombocytopenia, nausea and vomiting, or any other toxicities. Cisplatin was reduced by 50% if patients had grade 2 neurotoxicity, creatinine clearance of 45–59 ml/min, grade 4 nausea and vomiting or any other toxicities. Chemotherapy was withheld if patients had grade 3 or higher neurotoxicity, a creatinine clearance of less than 45 ml/min; neutrophil counts of less than 1500/mm^3^, platelet counts of less than 100,000/mm^3^, alanine aminotransferase or aspartate aminotransferase more than five times the upper limit of normal; or total bilirubin more than three times the upper limit of normal. Chemotherapy was terminated completely if adequate hematologic, renal, and liver function cannot be regained within two weeks of delay.

### 2.3 Radiotherapy

Radiotherapy was delivered one fraction daily for 5 days in a row per week to all the patients and the guidelines for planning and delivery of IMRT were based on previous reports.^3^, ^27^ In brief, all patients were fixed in a supine position with thermoplastic masks. CT scans with iopromide contrast using 3‐mm thick‐layer interval‐free scanning from the head to 1 cm below the sternoclavicular joints were conducted for planning. Target delineations were based on the concepts of ICRU reports 50 and 62. A total of 7000 cGy/30‐33F to the planning target volume (PTV) of the nasopharynx, 6600–6800 cGy/30‐33F to the PTV of enlarged lymph nodes, 6000 cGy/30‐33F to the high‐risk PTV and 5400 cGy/30‐33F to the low‐risk PTV were prescribed. Patients in the IC+RT arm commenced their radiotherapy 21 days after the first day of the second cycle of induction chemotherapy and patients in the CCRT arm commenced theirs at the first day of the first cycle of concurrent chemotherapy.

### 2.4 Evaluation of response and acute toxicities

At the end of radiotherapy and 3 months after, treatment responses were evaluated with flexible nasopharyngoscopy and MRI/CT scanning of the nasopharynx and neck. Treatment response assessment was conducted according to the Response Evaluation Criteria in Solid Tumors, version 1.1 (RECIST v.1.1).^28^ Acute toxicities were categorized and graded based on the National Cancer Institute Common Terminology Criteria for Adverse Events, version 3.0 (NCI‐CTCAE v. 3.0).^29^

### 2.5 Follow‐up

Patients underwent weekly evaluations during treatment and were assessed every 3 months during the first 2 years of follow‐up, then every 6 months thereafter. Each of the endpoints was assessed or confirmed by the physician in charge. Whenever possible, fine‐needle aspiration or biopsy was performed to confirm locoregional or distant relapse. Patients with documented relapse or persistent disease were provided with salvage treatments including re‐irradiation, chemotherapy, and surgery.

### 2.6 Statistical analysis

Our study is a prospective randomized non‐inferiority trial. A sample size of 194 with 97 to each arm was required when calculating on the Power and Sample Size Program with a power of 80%, a non‐inferiority margin of 10%, and a 2‐year PFS of 75% in the CCRT arm. We therefore needed to enrol a total of 204 patients with 102 to each arm, assuming a dropout or loss rate of 5%.

The primary end point of this study was PFS, and secondary end points included OS, LRRFS, DMFS, treatment response, and acute treatment toxicities. PFS was calculated from the date of randomization to documented disease progression (either locoregional recurrence or distant metastasis) or death from any cause, whichever occurred first. OS was calculated from the date of randomization to death from any cause or last follow‐up for patients still alive. LRRFS was calculated from the date of randomization to the first documented locoregional recurrence or last follow‐up for patients who had no locoregional failure. DMFS was calculated from the date of randomization to the first documented distant metastasis or last follow‐up for patients who had no distant failure.

All statistical analyses were done using SPSS (version 24.0) and Kaplan‐Meier survival curves were plotted with GraphPad Prism (version 8.0). All time‐to‐event survival rates and univariable analysis were performed with Kaplan–Meier method. The log‐rank test was performed to compare the survivals of different treatment arms. Treatment response, treatment toxicities, and other categorical variables were compared with the chi‐squared test (or Fisher's exact test, if indicated) and continuous variables were compared with the *t*‐test. All statistical tests were two‐sided, and a *p*‐values of 0.05 or less was deemed to indicate statistical significance.

## 3 RESULTS

**FIGURE 1:**
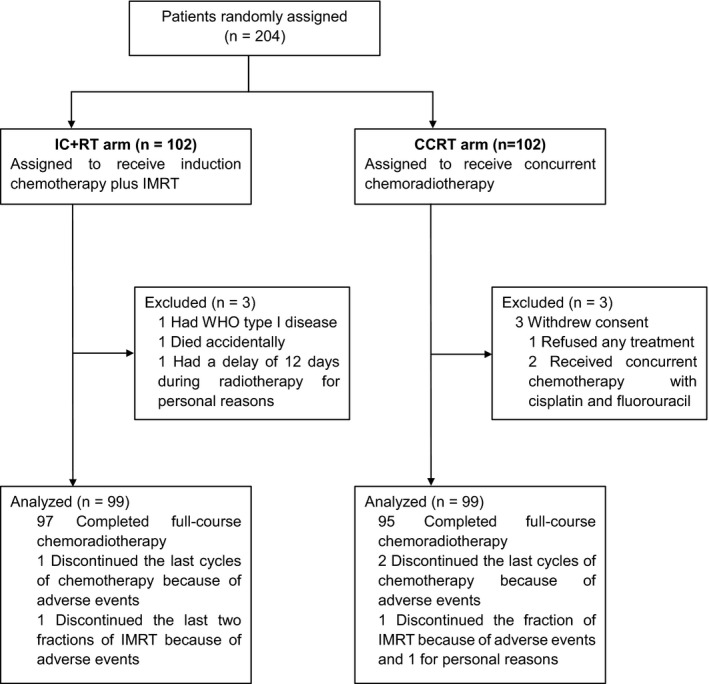
Flow chart of the trial. Note: Both the IC+RT arm and the CCRT arm received one cycle of docetaxel plus cisplatin and one cycle of cisplatin alone. Abbreviations: IC, induction chemotherapy; RT, radiotherapy; CCRT, concurrent chemoradiotherapy; IMRT, Intensity‐modulated radiotherapy

From June 27, 2013 to September 10, 2018, a total of 204 eligible patients were enrolled in the study, with 102 patients randomly assigned to each arm. Six patients, three in each arm, were excluded for the following reasons: one patient had WHO type I NPC; three patients withdrew consent including one patient refused any treatment and two patients did not receive the chemotherapy regimen as the protocol recommended; one patient in the IC+RT arm died accidentally just after the first cycle of induction chemotherapy and had incomplete treatment; and one patient violated the protocol and had a delay of 12 days during radiotherapy. Therefore, 99 patients (97%) in the IC+RT arm and CCRT arm respectively had received the assigned treatment and were evaluable for treatment response, outcomes, and toxicities (Figure 1). All patients were re‐staged according to the 8th edition of the UICC/AJCC staging system and the clinical characteristics of both treatment arms were well balanced (Table 1).

**TABLE 1:** Patient clinical characteristics

	IC+RT arm	CCRT arm	*p*‐value
Number	99	99	
Age, years			1
Median	51	50	
Range	22–68	24–68	
Gender			1
Male	75	75	
Female	24	24	
Smoker	60	68	0.234
Histology			0.523
WHO Type II	25	29	
WHO Type III	74	70	
T classification^a^			0.198
T1	10	20	
T2	14	16	
T3	50	44	
T4	25	19	
N classification^a^			0.728
N0	6	4	
N1	17	13	
N2	54	56	
N3	22	26	
Staging^a^			0.748
II	2	1	
III	53	57	
IVA	44	41	

Abbreviations: CCRT, concurrent chemoradiotherapy; IC, induction chemotherapy; RT, radiotherapy.

^a^ Patients were re‐staged according to the 8th edition of the UICC/AJCC staging system although only patients with stage III to IVB disease according to the 7th edition of the UICC/AJCC staging system were enrolled.

### 3.1 Treatment completion

Overall, 195 (98.5%) of 198 patients completed two cycles of TP/DDP chemotherapy, while the other 3 (1.5%) received only one cycle of TP chemotherapy due to adverse events. One patient with hepatitis B virus infection in the IC+RT arm discontinued the second cycles of chemotherapy because of febrile neutropenia and hepatoxicity. Two other cases of discontinuation in the CCRT arm were due to leukocytopenia. Two (2%) patients in the IC+RT arm and 4 (4%) in the CCRT arm (*n *= 4) had dose reductions of cisplatin by 25% for the second cycle of chemotherapy, mainly due to hematological toxicities.

One hundred ninety‐five (98.5%) out of 198 patients completed IMRT in conformity to the protocol. Because of grade 3 mucositis, 1 patient in the IC+RT arm and 2 in the CCRT arm quit the last 2 fractions of radiotherapy. These three patients all had comprehensive examinations including fiberscope and cervical ultrasound to ensure that they had achieved a complete response when discharged.

### 3.2 Treatment response

One hundred fifty‐five (78.3%) out of 198 patients achieved complete response. Thirteen (13.1%) out of 99 in the IC+RT arm and 9 (9.1%) out of 99 patients in the CCRT arm achieved a partial response in at the primary site. Among 188 patients with cervical lymph node involvement, 14 (15.1%) out of 93 in the IC+RT arm and 12 (12.6%) out of 95 in the CCRT arm achieved a partial response. At 3 months after treatment, 192 (97%) out of 198 patients had achieved complete response and with no residual primary lesions. At the same time, 5 (5.4%) out of 93 in the IC+RT arm and 1 (1.1%) out of 95 in the CCRT arm remained palpable and had positive ultra‐sound cervical lymph nodes. Salvage dissection was given to these six patients and the postoperative pathology remained positive except for one patient from the IC+RT arm. No significant difference was observed in treatment response between the two treatment arms both at the end of treatment or 3 months later (*p *> 0.05) (Table 2).

**TABLE 2:** Treatment response

	IC+RT arm	CCRT arm	*p*‐value
	*n* (%).	*n* (%).	
At the end of treatment			
Complete response	76 (76.8)	79 (79.8)	0.605
Persistent disease in primary site	13 (13.1)	9 (9.1)	0.366
Persistent disease in cervical nodes^a^	14 (15.1)	12 (12.6)	0.631
3 months after treatment			
Complete response	94 (94.9)	98 (99)	0.212
Persistent disease in cervical nodes^a^	5 (5.4)	1 (1.1)	0.116

Abbreviations: CCRT, concurrent chemoradiotherapy; IC, induction chemotherapy; RT, radiotherapy.

^a^ Data of treatment response in cervical nodes was analyzed based on 188 patients with cervical lymph node involvement, whereas the other was based on all 198 eligible patients. Treatment response was assessed according to RECIST v.1.1.

### 3.3 Survival outcome

At the last follow‐up date of June 25th 2020, the median follow‐up time was 45 months (range 4 to 84 months), with 56 (56.6%) patients in the IC+RT arm and 72 (72.7%) in the CCRT arm being followed up for at least three years and 11 (5.6%) patients were lost to follow up. Overall, 36 (17 in the IC+RT and 19 in the CCRT) patients died. Sixteen (9 in the IC+RT and 7 in the CCRT) and 30 (16 in the IC+RT and 14 in the CCRT) patients developed locoregional recurrences and distant metastasis, respectively, including 6 (3 in each arm) cases with both locoregional and distant failure. The difference in survival and failure patterns between the two arms were non significant (*p *> 0.05) (Table 3).

**TABLE 3:** Survival, living status and failure pattern

	IC+RT arm	CCRT arm	*p*‐value
Survival rate, %			
3‐year PFS rate	72.2	82.6	0.279
3‐year OS rate	87.8	92.8	0.911
3‐year LRRFS rate	92.3	94.7	0.652
3‐year DMFS rate	82.7	88.2	0.586
Living status, *n* (%)			0.295
Living	74 (74.7)	77 (77.8)	
Death	17 (17.2)	19 (19.2)	
Lost	8 (8.1)	3 (3)	
Failure pattern, *n* (%)	22 (22.2)	18 (18.2)	0.479
Locoregional	9 (9.1)	7 (7.1)	0.602
Distant	16 (16.2)	14 (14.1)	0.692
Locoregional and distant	3 (3)	3 (3)	1

Abbreviations: CCRT, concurrent chemoradiotherapy; DMFS, distant metastasis‐free survival; IC, induction chemotherapy; LRRFS, locoregional recurrence–free survival; OS, overall survival; PFS, progression‐free survival; RT, radiotherapy.

**FIGURE 2:**
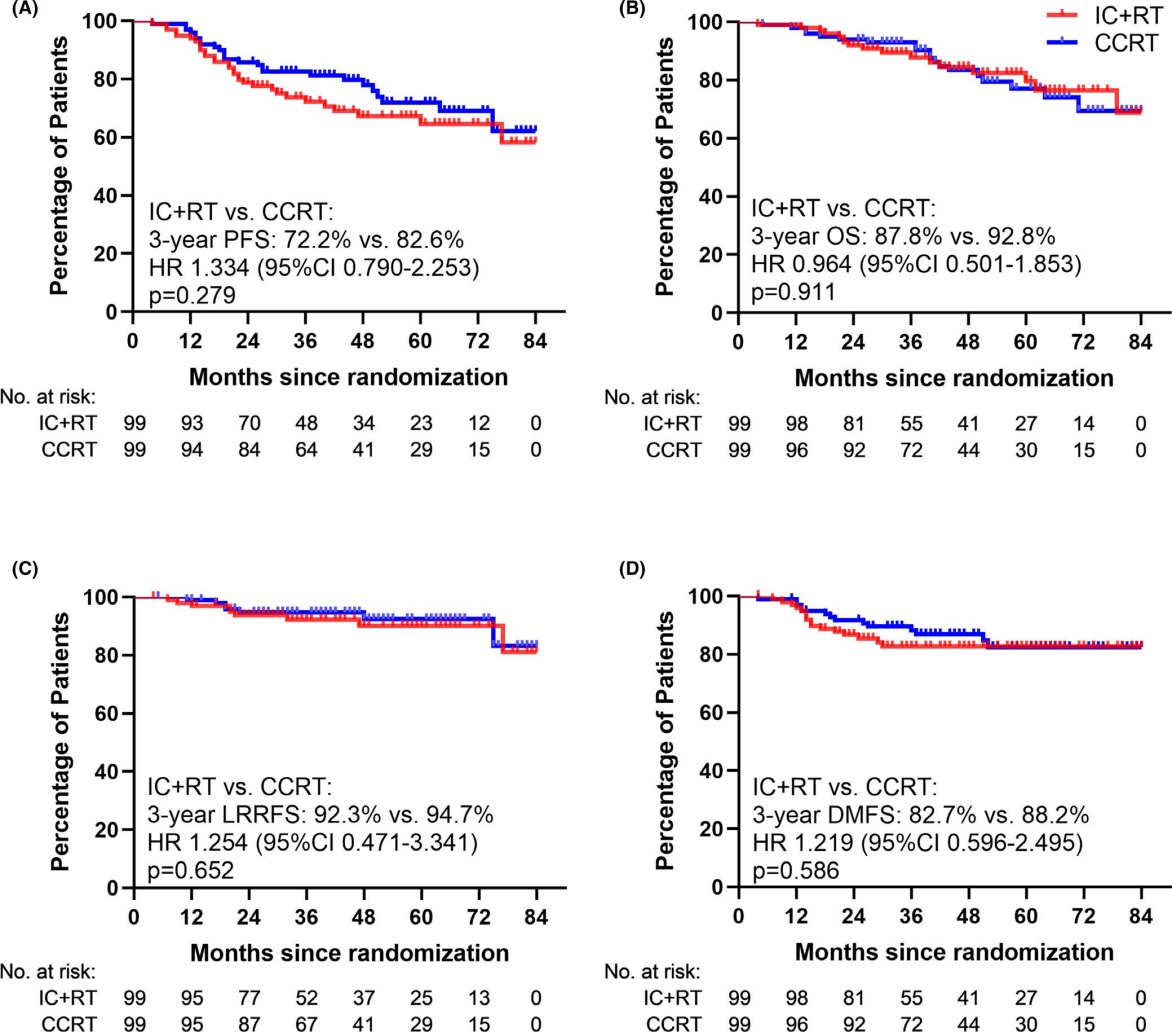
Kaplan‐Meier survival curves of two treatment arms

The 3‐year PFS, OS, LRRFS and DMFS were 72.2%, 87.8%, 92.3%, and 82.7% in the IC+RT arm, compared with 82.6%, 92.8%, 94.7%, and 88.2% in the CCRT arm. No statistical difference of in PFS, OS, LRRFS, or DMFS was observed between the two treatment arms, despite that the survival rates in the CCRT arm were slightly better than those in the IC+RT arm (*p *> 0.05) (Table 3, Figure 2). At the time of last follow‐up, the median PFS, OS, LRRFS, and DMFS had not been reached in both arms.

### 3.4 Treatment toxicities

There were no grade 5 adverse events or grade 4 non‐hematological adverse events observed in either arms. The incidences of leukopenia (*p *= 0.008) and anemia (*p *= 0.015) were significantly higher in patients of the CCRT arm than those of the IC+RT arm, while the incidences of neutropenia, thrombocytopenia and non‐hematological toxicities were similar in both arms (*p *> 0.05) (Table 4).

**TABLE 4:** Treatment toxicities

	IC+RT arm				CCRT arm				*p*‐value
	*n* (%)				*n* (%)				
	Grade 1	Grade 2	Grade 3	Grade 4	Grade 1	Grade 2	Grade 3	Grade 4	
Hematological									
Leukopenia	13 (13.1)	28 (28.3)	27 (27.3)	8 (8.1)	9 (9.1)	35 (35.4)	37 (37.4)	12 (12.1)	0.008
Neutropenia	5 (5.1)	12 (12.1)	13 (13.1)	44 (44.4)	8 (8.1)	20 (20.2)	17 (17.2)	42 (42.4)	0.098
Anemia	46 (46.5)	25 (25.3)	9 (9.1)	0 (0)	55 (55.6)	20 (20.2)	10 (10.1)	6 (6.1)	0.015
Thrombocytopenia	20 (20.2)	1 (1)	1 (1)	0 (0)	22 (22.2)	2 (2)	0 (0)	0 (0)	0.846
Nonhematological									
Skin desquamation	55 (55.6)	22 (22.2)	3 (3)	‐	45 (45.5)	27 (27.3)	7 (7.1)	‐	0.371
Mucositis	41 (41.4)	38 (38.4)	5 (5.1)	‐	47 (47.5)	31 (31.3)	6 (6.1)	‐	0.751
Oral fungal infection	20 (20.2)				20(20.2)				1
Nausea and vomiting	16 (16.2)	13 (13.1)	1 (1)	‐	20 (20.2)	13 (13.1)	1 (1)	‐	0.899

Note

Treatment toxicities were categorized and graded according to the NCI CTCAE v.3.0.

Abbreviations: CCRT, concurrent chemoradiotherapy; IC, induction chemotherapy; RT, radiotherapy.

### 3.5 Univariable analysis and subgroup analysis

Univariable analysis was conducted to detect prognostic predictors in this trial. The potential prognostic factors included treatment arm, gender, smoking, age, and overall clinical stage. Two patients in the IC+RT arm and 1 in the CCRT arm with stage II disease according to the 8th edition of the UICC/AJCC staging system were excluded when univariable analysis was based on clinical stage. As a result, clinical stage was found to be the independent predictor for PFS (*p *= 0.01) and OS (*p *= 0.005). The other variables were not found prognostic factors for any event‐free survival (*p *> 0.05) (Table 5). Subgroup analysis revealed that in subgroups with stage III and IVA disease according to the 8th edition of the UICC/AJCC staging system and subgroup excluding patients with low risk of distant metastasis (those with no nodal involvement^30^), no significant difference of any event‐free survival was observed between the two treatment arms (*p*>0.05) (Table 6).

**TABLE 5:** Univariable analysis

	3‐year PFS rate	3‐year OS rate	3‐year LRRFS rate	3‐year DMFS rate
IC+RT vs CCRT, %	73.7 vs. 82.8	86.9 vs. 92.5	95.1 vs. 94.4	84.7 vs. 90.2
*p*‐value	0.445	0.932	0.842	0.526
Male vs Female, %	73.3 vs. 91.6	87.5 vs. 100	92.8 vs. 95.8	83.6 vs. 91.6
*p*‐value	0.08	0.207	0.886	0.304
Smoker vs Nonsmoker, %	75.1 vs. 82.2	90.2 vs. 90.9	94.3 vs. 92.1	83.3 vs. 89.6
*p*‐value	0.4	0.805	0.598	0.519
<60y vs ≥60y,%	75.4 vs. 86.5	90.7 vs. 89.2	92.7 vs. 97.3	82.8 vs. 97.3
*p*‐value	0.697	0.645	0.958	0.073
stage III vs. stage IV^a^, %	85.1 vs. 68.8	94.4 vs. 96.2	95.2 vs. 92.6	89.7 vs. 79.8
*p*‐value	0.01	0.005	0.237	0.109

Abbreviations: CCRT, concurrent chemoradiotherapy; DMFS, distant metastasis‐free survival; IC, induction chemotherapy; LRRFS, locoregional recurrence–free survival; OS, overall survival; PFS, progression‐free survival; RT, radiotherapy.

^a^ 2 patients in the IC+RT arm and 1 in the CCRT arm with stage II disease according to the 8th edition of the UICC/AJCC staging system were excluded when univariable analysis was based on clinical stage.

**TABLE 6:** Subgroup analysis

	IC+RT arm	CCRT arm	*p*‐value
Survival rate of stage III, %			
3‐year PFS rate	77	92.8	0.111
3‐year OS rate	92.2	96.5	0.869
3‐year LRRFS rate	89.9	100	0.053
3‐year DMFS rate	86.7	92.8	0.613
Survival rate of stage IVA, %			
3‐year PFS rate	68.3	68.2	0.607
3‐year OS rate	84.1	87.7	0.601
3‐year LRRFS rate	97.7	86.9	0.139
3‐year DMFS rate	78.2	80.9	0.972
Survival rate of N+ disease, %			
3‐year PFS rate	77	82.1	0.858
3‐year OS rate	88.6	92.2	0.511
3‐year LRRFS rate	94.7	94.1	0.876
3‐year DMFS rate	86.2	89.8	0.802

Abbreviations: CCRT, concurrent chemoradiotherapy; DMFS, distant metastasis‐free survival; IC, induction chemotherapy; LRRFS, locoregional recurrence–free survival; OS, overall survival; PFS, progression‐free survival; RT, radiotherapy.

## 4 DISCUSSION

To our best knowledge, this is the first randomized non‐inferiority trial to compare the efficacy and toxicities of IC plus RT and CCRT in patients with LA‐NPC treated using IMRT. Our results suggest that sequential TP/DDP IC followed by IMRT alone and CCRT yielded similarly favorable treatment outcomes in terms of PFS, OS, LRRFS, and DMFS, but with a lower incidence of hematological toxicities for patients with LA‐NPC.

CCRT with a platinum‐based regimen has been considered the standard treatment for LA‐NPC as the value of additional chemotherapy given concurrently with conventional radiotherapy has been repeatedly shown by randomized trials^4^, ^5^, ^6^, ^7^, ^8^, ^9^, ^10^ as well as meta‐analyses.^11^, ^12^, ^13^ However, the application of IMRT improves locoregional control, which may diminish the benefit of concurrent chemotherapy in LA‐NPC. A retrospective study by Su et al. reported that, compared to IMRT alone, combining chemotherapy and IMRT failed to prolong the 5‐year PFS, OS, LRRFS, and DMFS.^14^ Another retrospective study also indicated that patients with LA‐NPC had similar 5‐year PFS, OS, LRRFS, and DMFS when treated by IMRT with simultaneous integrated boost or CCRT.^15^

A network meta‐analysis including 27 studies and 7940 patients showed IC plus CCRT provided the best PFS, OS, and DMFS in LA‐NPC compared to CCRT and CCRT plus AC.^16^ The long‐term results of a phase III randomized trial demonstrated that the addition of IC to CCRT significantly prolonged the 5‐year PFS, LRRFS, DMFS, and OS for patients with LA‐NPC.^17^, ^18^ However, up to 72.8% of patients in the IC plus CCRT group developed grade 3 or 4 toxicities compared to 53.8% in the CCRT group. Only 88% and 30.3% patients in the IC plus CCRT group completed all three cycles of IC and concurrent chemotherapy, respectively. Similarly, Zhang et al. reported another phase III randomized trial comparing gemcitabine and cisplatin (GP) IC followed by CCRT versus CCRT alone.^19^ The authors revealed that combination of GP IC and CCRT offered significant improvement in 3‐year PFS, OS, and DMFS. Again, acute toxicities were significantly higher in the IC plus CCRT group. These reports demonstrated the great significance of the IC followed by CCRT approach in the management of LA‐NPC. However, increased acute toxicities, additive cost, and reduced compliance also gave rise to much concern.

Although there was no prospective randomized trial published investigating the therapeutic value of IC followed by IMRT, retrospective studies and meta‐analyses had shown high efficacy with low toxicities^20^, ^21^, ^22^, ^23^, ^24^ in patients with LA‐NPC. Lin et al. suggested that IC plus IMRT resulted in superb 3‐year PFS, OS, LRRFS, and DMFS.^20^ Furthermore, their results showed that additive concurrent chemotherapy offered no significant improvement in survival but increased grade 3 or 4 acute toxicities. Li et al. reported a propensity‐matched analysis including 147 patients with 49 in each group to compare IC plus IMRT, CCRT, and IC plus CCRT for LA‐NPC.^21^ Their study revealed that these different modalities provided comparable treatment outcomes and acute toxicities. Another propensity‐matched study retrospectively analyzing 396 patients to investigate the efficacy of IC plus IMRT and CCRT in LA‐NPC also observed no significant survival differences.^23^ Likewise, Qiu et al. found similar outcomes in terms of 5‐year PFS, OS, LRRFS, and DMFS between 117 patients who underwent IC plus IMRT and 123 patients receiving CCRT plus AC, but IC plus IMRT significantly reduced the incidence of grades 3 or 4 nausea–vomiting and leukopenia.^24^ A meta‐analysis analyzing 8 studies and 2605 patients showed IC plus RT achieved similar PFS, OS, LRRFS, and DMFS as IC plus CCRT and resulted in less hematological toxicities during radiation.^22^ Their subgroup analysis revealed survival outcomes remained similar in subgroups with or without two‐dimensional radiotherapy.

In our present study, the 3‐year PFS, OS, LRRFS, and DMFS were comparable between IC+RT and CCRT. Both arms achieved similarly favorable treatment outcomes, which was consistent with previous retrospective studies.^20^, ^21^, ^23^, ^24^ Notably, both treatment arms produced a particularly good outcome of LRRFS, which supports the hypothesis that the significant improvement in locoregional control resulting from the application of IMRT may diminish the value of concurrent chemotherapy in LA‐NPC. In this study, 16 patients developed distant metastasis and 9 patients developed locoregional recurrences in the IC+RT arm, compared to 14 and 7 patients in the CCRT arm, respectfully. Distant metastasis remained the predominate pattern of treatment failure, but chemotherapy given concurrently failed to prolong DMFS. Previous phase III randomized trials demonstrated that IC‐CCRT significantly improved DMFS and therefore improved OS compared to CCRT.^17^, ^18^, ^19^ Nevertheless, with the combined utilization of IC and IMRT, whether concurrent chemotherapy plays a principle role in the improvement of DMFS and OS remains unknown. Our study suggested that the combination of sequential IC followed by IMRT alone resulted in high efficacy, and may be considered as an alternative to concurrent chemotherapy. We also observed more frequent and severer leukopenia and anemia in the CCRT arm than in the IC+RT arm, which was consistent with previous retrospective studies.^20^, ^24^

The chemotherapy regimen in our present study protocol was administered as one cycle of TP (docetaxel 75 mg/m^2^ on day 1 and cisplatin 25 mg/m^2^ per day from day 1 to 3) and one cycle of DDP alone (cisplatin 25 mg/m^2^ per day from day 1 to 3). DDP in our present study protocol was much less intense than in the standard chemotherapy regimen adopted in most studies, which was concurrently administered as cisplatin 100 mg/m^2^ on a single day every 3 weeks for three cycles. However, our 3‐year survival outcomes in the CCRT arm were comparable to those prospective randomized trials adopting standard chemotherapy regimen. Sun et al. published a 3‐year PFS, OS, LRRFS, and DMFS of 72%, 86%, 89%, and 83% in the CCRT group.^17^ Likewise, Zhang et al. reported a 3‐year PFS, OS, LRRFS, and DMFS of 76.5%, 90%, 91%, and 84% in the CCRT group.^19^ These two trials both enrolled node‐positive stage III–IVB NPC (7th edition of the UICC/AJCC). Correspondingly, our 3‐year PFS, OS, LRRFS, and DMFS were 81.9%, 92.5%, 94.4%, and 87.7% in the CCRT arm after excluding N0 disease. Similar survival outcomes in patients accepting either standard chemotherapy or our TP/DDP chemotherapy protocol indicated that low dose chemotherapy may be feasible for a large majority of LA‐NPC cases treated with IMRT. It would be more meaningful if molecular biomarkers data could be included to further identify patients who would have the most benefit from low intensity approaches.

Owing to the longtime span of patient enrollment, 2013–2018, we considered plasma Epstein‐Barr virus DNA data might not adequate in this study because of poor comparability. It should also be noted that this is a phase II, single center trial with relatively small sample size which might have inadequate power to detect differences between the two strategies. Chemotherapy dose in this study was relatively low compare to NCCN guideline. De‐density treatment without compromise of clinical endpoints is our goal to achieve. Even though this study showed promising result at 3 years and dozens of cases in both arms had reached 5 years survival, more patients and further investigations to search biomarkers for possible beneficial candidate remained as our future mission.

In conclusion, the results of this trial suggest that IC followed by IMRT alone provided good PFS, OS, LRRFS, and DMFS, comparable to CCRT but with lower incidences of hematological toxicities for patients with LA‐NPC. IC plus IMRT is a promising option and deserves to be further confirmed by long‐term follow‐up and multicenter large‐scale prospective trials.

## CONFLICT OF INTEREST

The authors declare no conflict of interest.

## AUTHOR CONTRIBUTIONS

Zhixiong Lin, Mei Li, and Zhining Yang were involved in all aspects of the study concept and design, drafting of the manuscript, and manuscript revision, and were the primary care providers for patients included in the study. Zeman Cai contributed to data collection, analysis, and interpretation, drafting of the manuscript, and manuscript submission and revision. Qingxin Cai, Yingji Hong, Cuidai Zhang and Kaichun Huang contributed to data collection and analysis. All authors were involved in any revision stages and approved the final version.

## CLINICAL TRIAL INFORMATION

This trial was registered at the Chinese Clinical Trial Registry with the registration number of ChiCTR‐TRC‐14004341.

## Data Availability

Because of patient's privacy and confidentiality of data, the data that support the findings of our study are only available from the corresponding author upon reasonable request.
